# Implementation of an active case management network to identify HIV‐positive infants and accelerate the initiation of antiretroviral therapy, Thailand 2015 to 2018

**DOI:** 10.1002/jia2.25450

**Published:** 2020-02-27

**Authors:** Rangsima Lolekha, Patcharaporn Pavaputanon, Thanyawee Puthanakit, Michael Martin, Pope Kosalaraksa, Witaya Petdachai, Thitiporn Borkird, Rawiwan Hansudewechakul, Archawin Rojanawiwat, Sarawut Boonsuk, Tanawan Samleerat, Sumet Ongwandee, on behalf of the ACC working group

**Affiliations:** ^1^ Division of Global HIV and TB Program Thailand/Asia Regional Office U.S. Centers for Disease Control and Prevention Thailand Office Nonthaburi Thailand; ^2^ Bureau of AIDS, TB and STIs Ministry of Public Health Nonthaburi Thailand; ^3^ Department of Pediatrics Faculty of Medicine Chulalongkorn University and HIVNAT Thai Red Cross AIDS Research Center and Center of Excellence in Pediatric Infectious Diseases and Vaccines Chulalongkorn University Bangkok Thailand; ^4^ Department of Pediatrics Faculty of Medicine Khon Kaen University Khon Kaen Thailand; ^5^ Prachomklao Hospital Petchaburi Thailand; ^6^ Hat Yai Hospital Songkla Thailand; ^7^ Chiang Rai Prachanukroh Hospital Chiang Rai Thailand; ^8^ Department of Medical Sciences Ministry of Public Health Nonthaburi Thailand; ^9^ Department of Health Ministry of Public Health Nonthaburi Thailand; ^10^ Department of Medical Technology Faculty of Associated Medical Sciences Chiang Mai University Chiang Mai Thailand

**Keywords:** HIV infection, active case management, HIV PCR, infants, antiretroviral treatment initiation

## Abstract

**Introduction:**

Early initiation of antiretroviral therapy (ART) can reduce HIV‐related morbidity and mortality in HIV‐positive infants. We implemented an Active Case Management Network to promote early ART initiation Aiming for Cure (ACC) in August 2014. We describe ACC implementation, early infant diagnosis (EID) coverage and ART initiation during August 2014 to July 2018 compared with a national EID survey during October 2007 to September 2011 (pre‐ACC).

**Methods:**

Thailand's 2014 HIV Treatment Guidelines recommend that HIV‐exposed infants have HIV polymerase chain reaction (PCR) testing at birth, one month and at two to four months. Testing is done at 14 national HIV PCR laboratories. When an HIV‐positive infant (HIV PCR+) is identified, PCR laboratory staff send the result to the hospital staff responsible for the infant's care and to the national laboratory case manager (CM). As part of ACC, the national laboratory CM alerts a regional CM who contacts the hospital staff caring for the infant to offer technical support with ART initiation and ART adherence. CMs enter clinical, demographic and laboratory data into the national ACC database. We analysed the ACC data from August 2014 to July 2018 to assess the ACC's impact on EID coverage, ART initiation and time‐to‐ART initiation.

**Results:**

The uptake of EID increased from 64% (pre‐ACC) to >95% in 2018 (ACC). The number of HIV‐positive infants born declined from 429 cases (pre‐ACC) to 267 cases (ACC). Median age at the first‐positive PCR declined from 75 days (pre‐ACC) to 60 days (ACC); *P* < 0.001. Among 429 infants diagnosed before ACC was started, 241 (56%) received ART; during ACC, 235 (88%) of 267 HIV‐positive infants received ART. The median age at ART initiation declined from 282 days before ACC to 83 days during ACC (*P* < 0.001) and the median time from blood collection to ART initiation declined from 168 days before ACC to 23 days during ACC (*P* < 0.001).

**Conclusions:**

An innovative case management network (ACC) has been established in Thailand and results suggest that the network is promoting EID and early ART initiation. The ACC model, using case‐managed PCR notification and follow‐up, may speed ART initiation in other settings.

## 1 Introduction

Approximately 700,000 infants are born in Thailand each year and about 4000 of these infants are born to HIV‐positive mothers 1. HIV infection of infants may occur in utero, during delivery or during breast feeding. Without antiretroviral therapy (ART), more than half of HIV‐positive infants estimated to die by their second birthday. The early initiation of ART reduces HIV‐related morbidity and mortality among infants infected with HIV 2, 3, 4. In addition, early ART may limit the establishment and the size of the HIV reservoir, potentially preserving immune function and providing an opportunity to achieve HIV remission 4. In at least one child, early ART was with a long period of viral suppression after ART was discontinued 4, 5.

The World Health Organization (WHO) recommends HIV testing of HIV‐exposed infants using virological tests including HIV DNA or RNA polymerase chain reaction (PCR) at four to six weeks of age, early infant diagnosis (EID) 6, and that ART be initiated if the PCR test result is positive regardless of CD4 count or WHO clinical stage. WHO also recommends a second PCR test to confirm the initial‐positive virological test result. However, many resource‐limited countries face challenges implementing EID and linking HIV‐positive infants to ART services 7, 8. Data from a national EID survey in Thailand in 2008 to 2011 9, 10 showed that 67% of infants born to HIV‐positive mothers received HIV EID within one year of birth 10. Only 12% of HIV‐positive infants in Thailand received ART before the age of six months, and 37% before one year 9, 10.

**Figure 1:**
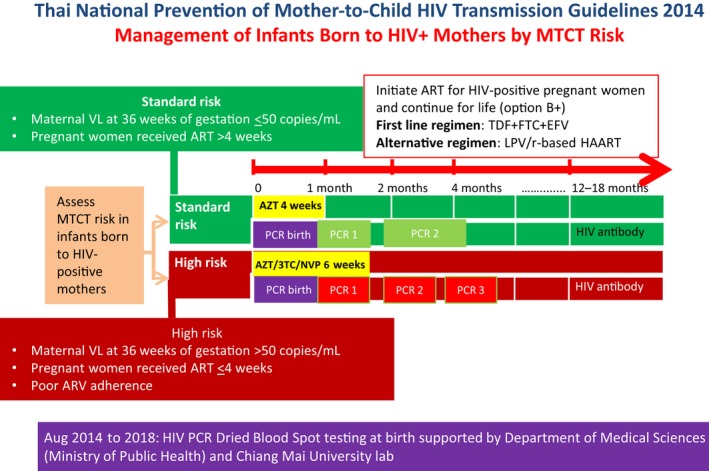
Thai national prevention of mother‐to‐child HIV transmission, 2014. 3TC, lamivudine; ART, antiretroviral therapy; AZT, azidothymidine; EFV, efavirenz; FTC, emtricitabine; LPV/r, lopinavir/ritonavir; MOPH, Ministry of Public Health; MTCT, Mother‐to‐child Transmission; NVP, nevirapine; PCR, polymerase chain reaction; TDF, tenofovir disoproxil fumarate; VL, viral load; wks, weeks.

Thailand's 2014 HIV Prevention, Care and Treatment Guidelines recommend that ART‐naïve HIV‐positive pregnant women should initiate antepartum ART‐containing tenofovir disoproxil fumarate plus lamivudine (3TC) or emtricitabine plus efavirenz regardless of CD4 cell count or clinical staging as soon as HIV is diagnosed and continue for life regardless of CD4 count. Lopinavir/ritonavir is recommended for the HIV‐positive pregnant woman suspected to have non‐nucleoside reverse‐transcriptase inhibitor resistance 11. HIV‐exposed infants are classified based on their risk of acquiring HIV. Infants at high risk (i.e. maternal plasma HIV viral load >50 copies/mL near the time of delivery (34 to 36 weeks gestational age) or infants born to mothers taking ART for less than four weeks before delivery) receive azidothymidine (AZT), 3TC and nevirapine for six weeks and HIV DNA PCR tests at birth, age one, two and four months (Figure 1). Infants at standard risk (i.e. an infant whose mother has documentation of plasma HIV viral load <50 copies/mL near the time of delivery. If maternal viral load is not available, the infant is considered standard risk if the mother has been taking ART for at least four weeks prior to delivery and has a history of good adherence) receive AZT for four weeks and HIV PCR at birth, age one and two to four months. The guidelines recommend starting all HIV‐positive infants on ART as soon as possible after diagnosis regardless of symptoms or CD4 count and exclusive infant formula feeding for all infants born to HIV‐positive mothers.

**Figure 2:**
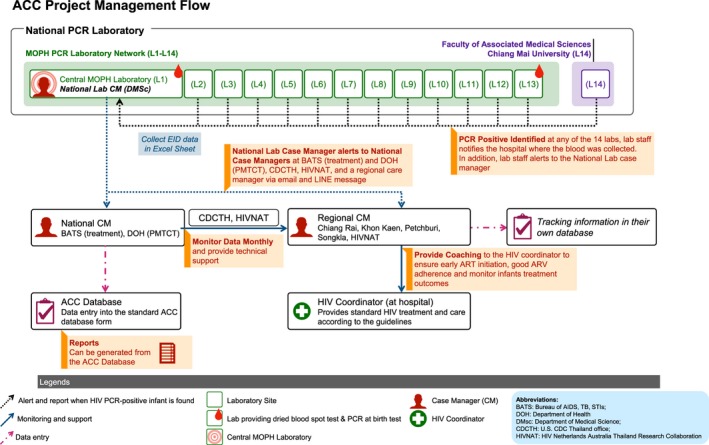
ACC Project management flow.

In order to achieve UNAIDS 90–90–90 targets to end the AIDS epidemic by 2020 12, the Thailand Ministry of Public Health (MOPH) collaborated with partners to develop and implement an Active Case Management Network to promote EID and early ART initiation among HIV‐positive infants Aiming for Cure project (ACC) (Figure 2) in August 2014. The ACC network aimed to increase coverage of EID among infants born to HIV‐positive mothers to 90% and link 90% of diagnosed HIV‐positive infants to ART as soon as possible after diagnosis 5. In this manuscript, we describe the national implementation of ACC and compare EID coverage and the time from HIV diagnosis to ART initiation during ACC with baseline data from the 2008 to 2011 national EID survey (pre‐ACC).

## 2 Methods

### 2.1 Establishment of the active case management network

In 2014, the Thailand MOPH (Department of Disease Control, Department of Health, Department of Medical Sciences) collaborated with the U.S. Centers for Disease Control and Prevention, Thailand office (CDC); HIV Netherlands Australia Thailand Research Collaboration (HIV‐NAT); Chiang Mai University, Department of Medical Technology; and four tertiary care hospitals in four regions of Thailand to create the ACC programme to promote EID using HIV PCR testing among HIV‐exposed infants and early ART initiation among HIV‐positive infants. ACC case managers (CMs) at hospital, regional and national levels provided technical assistance, coaching and management training to hospital‐based multi‐disciplinary teams to promote EID, prompt ART initiation and to improve the quality of prevention of mother‐to‐child transmission (PMTCT) services. We established a group on the social media app LINE for real‐time communication between CMs, and developed and distributed job aids to all public hospitals to provide guidance on the clinical management of challenging PMTCT and paediatric HIV cases.

In August 2014, the MOPH implemented HIV PCR dried blood spot (DBS) testing of infants born to HIV‐positive mothers at birth regardless of MTCT risk as part of the ACC project (August 2014 to 2017). In 2018, PCR at birth was included in the benefit package of the national AIDS programme for high MTCT risk babies 13. PCR at birth testing is defined as PCR testing of infants <7 days old. Fourteen laboratories in Thailand (Figure 2) provide HIV PCR testing for EID free of charge to Thai citizens under Thailand's national health insurance programme which covers more than 95% of HIV PCR testing in Thailand. Non‐Thai citizens can access free EID using a migrant health insurance card or a social welfare fund (i.e. the Princess Soamsawali PMTCT Fund (2014 to 2018), other hospital social welfare funds).

### 2.2 ACC project management flow

When laboratory staff identify an HIV PCR‐positive result in an infant born to an HIV‐positive mother at one of the 14 HIV PCR laboratories, the staff person notifies the hospital HIV coordinator where the blood was collected by surface mail and using an online National AIDS Program database (Figure 2). Details of the EID test platform and information on blood sample collection of the national EID programme can be found elsewhere 10, 14. Before ACC, there was no routine reporting system to acknowledge receipt of the notification. As part of ACC a routine reporting system was established. When an HIV PCR‐positive result occurs, laboratory staff members alert the national laboratory CM. The national laboratory CM collects PCR test result information, enters results in the national ACC database, and notifies the national PMTCT CM, the national treatment CM, and the responsible regional CM by email and LINE message, receiving an acknowledgement upon receipt of the notification. Then, the regional CM contacts the HIV coordinator at the hospital where the case was identified to ensure prompt ART initiation. Before ACC implementation in 2014, the treatment criteria for ART initiation of HIV‐positive infants was two positive PCR results (i.e. confirmed HIV infection) or one positive PCR result without a conflicting PCR result (i.e. probable HIV infection). To encourage prompt ART initiation during ACC implementation, the national ACC network recommended that hospital staff initiate ART as soon as possible after the first‐positive PCR test result. For example on the day that caregivers bring the infant back for a follow‐up visit, blood can be collected for the second PCR to confirm diagnosis, and ART initiated. The regional CM also provides ART adherence support to HIV‐positive infants and caregivers according to the national HIV treatment and care guideline (Figure 1).

Hospital HIV coordinators collect standardized information about HIV‐positive infants including CD4 count, viral load, the date the infant‐initiated ART, and the mother's history of PMTCT and send the findings to the regional CM. Regional CMs send data to the national treatment CM who is responsible for cleaning and entering the data into the national ACC database web application (version 2016; develop by Visual Studio 2008 Professional Edition at http://cqihiv.com/pedcare/), and preparing monthly ACC reports. National and regional CMs access ACC reports online and use them for continuous quality improvement. No personal identifying information is provided in social media posts or in email communications.

**Figure 3:**
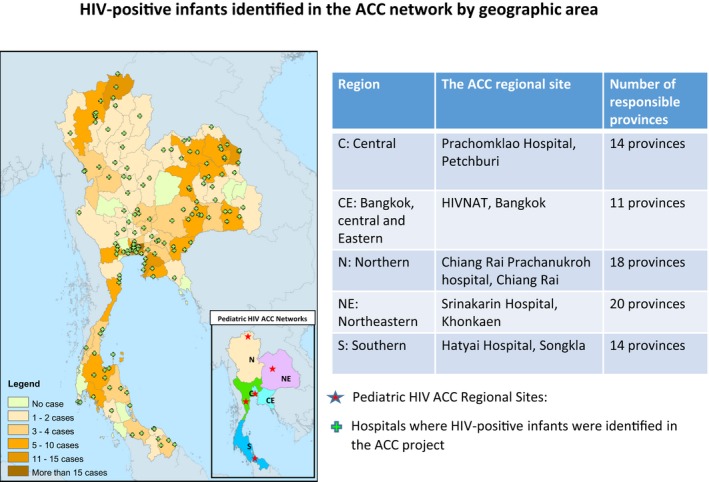
HIV‐positive infants identified in the ACC network by geographical area.

Regional CMs are responsible for hospitals in 11 to 20 provinces (Figure 3). The national treatment CM provides support to regional CMs to ensure national HIV treatment guidelines are implemented. The national PMTCT CM works with regional CMs and hospitals reporting new perinatal infection cases to identify causes of new perinatal HIV infections and ensure that PMTCT guidelines are implemented.

### 2.3 Data collection and analysis

The national ACC programme collects data from all infants who have a positive HIV PCR test result at 14 participating laboratories. The data collected include: date of the positive HIV PCR result, date the national laboratory and regional CMs were notified, type of hospital (e.g. university, government, provincial, district, etc.), province that sent the HIV PCR specimen, type of specimen, infant characteristics (e.g. gender, nationality, date of birth, age at first PCR test), history of maternal PMTCT services, and infant's treatment outcome (e.g. birth weight, CDC HIV clinical stage, CD4 count, VL result). Informed consent for data collection was not obtained because the data are routinely collected and investigators did not have contact with human subjects or access to personal identifiers. We used two levels of security to protect participant's privacy. A unique ACC code was automatically generated by the ACC database when the national laboratory CM entered data of a newly diagnosed HIV‐positive infants into the database, and this code was used to link data. The ACC code cannot be linked to personal identifiers. Second, access to the ACC database is password protected for authorized investigators.

The number of HIV‐positive infants, type of specimen, age at PCR blood collection, coverage of ART and age at ART initiation during 2015 to 2018 ACC (August 2014‐July 2018; latest update data as of August 2018) were compared with data from the 2008 to 2011 national EID survey (October 2007 and September 2011), before ACC, using Chi‐square test. The median age of blood collection and ART initiation and time between HIV diagnosis and ART initiation were compared using Wilcoxon rank sum test.

We used EID uptake data (2016 to 2018) from Thailand's National Global AIDS Monitoring (GAM) reports and UNAIDS reports 2016 to 2018 1, 15, 16. To calculate the MTCT rate, we used the number of infants with confirmed PCR positive in the ACC as a numerator and the number of infants who had PCR tests done at the 14 PCR laboratories (reported in the GAM report 2015 to 2018) as the denominator 1.

The Ethical Review Committee of the Thailand MOPH reviewed and approved the study protocol. The protocol was also reviewed in accordance with CDC human research protection procedures and determined to be a nonresearch programme evaluation activity.

## 3 Results

The national ACC programme was launched in August 2014. Regional CMs oversaw case notifications from 900 hospitals 17 that submitted EID specimens to the 14 participating laboratories from all 77 provinces of Thailand. From August 2014 to July 2018, 267 HIV‐positive infants were identified from 150 hospitals in 69 provinces. A total of 69 (26%) cases were reported from 36 hospitals in 12 provinces included in the Bangkok area and Eastern region, 63 (24%) cases from 38 hospitals in 17 provinces in the Northeastern region, 50 (19%) cases from 24 hospitals in 12 provinces in the Central region, 47 (18%) cases from 27 hospitals in 17 provinces in the Northern region and 38 (14%) cases from 25 hospitals in 11 provinces in the Southern region (Figure 3).

The uptake of EID increased from 64% during 2008 to 2011 10 to 73% of HIV‐exposed infants in 2013 17, before the launch of the ACC, to 92% in 2016, 94% in 2017 and >95% in 2018 15, 16, 18. The number of HIV‐positive infants born declined from 429 during 2008 to 2011 (pre‐ACC) to 267 during 2015 to 2018 (ACC). During 2008 to 2011, DBS were used to diagnose 41% of the EID‐positive specimens; this increased to 70% during the 2015 to 2018 with ACC activities (*P* < 0.001) (Table 1).

**Table 1:** Characteristics and ART use of HIV PCR‐positive infants identified by the Active Case Management Network (ACC), 2015 to 2018 and the national Early Infant Diagnosis Survey (EID), 2008 to 2011, Thailand

	EID 2008 to 2011^a^ (n = 429) N (%)	ACC 2015 to 2018^b^ (N = 267) N (%)	*P*‐value
Number of PCR + infants			
Central laboratory	176 (41.0)	117 (43.8)	<0.001^c^
Regional laboratories	174 (40.6)	54 (20.2)	
Chiang Mai Medical Technology laboratory	79 (18.4)	87 (32.6)	
Other laboratories	0 (0)	9 (3.4)	
Type of specimen			
Whole blood	253 (59.0)	59 (22.1%)	<0.001^c^
Dried blood spot	176 (41.0)	187 (70.0%)	
Missing data	0 (0)	21 (7.9%)	
Sex of infant			
Male	N/A	123 (46.1%)	–
Female		144 (53.9%)	
Nationality			
Thai	N/A	249 (93.3%)	–
Non‐Thai		18 (6.7%)	
Born to high MTCT risk mom	N/A	227 (85.0%)	–
Age blood collected for first‐positive PCR (days)			
Median (IQR Range)	75 (55 to 124)	60 (29 to 133)	
At birth^d^ (0 to 7 days)		60 (22.5%)	<0.001^c^
<1 month (7 to 29 days)	19 (4.4)	13 (4.9)	
1 to 2 months (30 to 59 days)	107 (24.9)	63 (23.6)	
2 to 3 months (60 to 89 days)	139 (32.4)	35 (13.1)	
3 to 4 months (90 to 119 days)	49 (11.4)	16 (6.0)	
4 to 5 months (120 to 149 days)	49 (11.4)	25 (9.4)	
5 to 6 months (150 to 179 days)	18 (4.2)	11 (4.1)	
≥6 months (≥180 days)	48 (11.2)	44 (16.5)	
Started ART^e^ (%)	241 (56.2)	235 (88.0)	<0.001^c^
Age ART initiation (days)			
Median (IQR)	282 (191 to 451)	83 (48 to 160)	
<1 month	1 (0.002)	27 (10.1)	<0.001^c^
1 to 2 months	0 (0)	54 (20.2)	
2 to 3 months	7 (1.6)	50 (18.7)	
3 to 4 months	12 (2.8)	21 (7.9)	
4 to 5 months	13 (3.0)	20 (7.5)	
5 to 6 months	19 (4.4)	12 (4.5)	
6 to 12 months	106 (24.7)	30 (11.2)	
≥12 months	83 (19.3)	21 (7.9)	
Missing	188 (43.8)	32 (12.0)	
Time from blood collection of first‐positive PCR to starting ART (days)			
	N = 241	n = 227	
Median (IQR)	168 (95 to 364)	23 (15 to 34)	<0.001^f^

N/A, not known or data not collected.

a 2008 to 2011: October 2007‐September 2011;

b 2015 to 2018: August 2014 to July 2018;

c chi‐square test;

d mean age of PCR at birth was 2.5 days (IQR: 2 to 3);

e started ART by the time of data analysis: EID (2012); ACC (As of August 2018);

f Wilcoxon rank sum test.

During August 2014‐July 2018, specimens were collected from 15,951 HIV‐exposed infants 1; 267 (1.7%) infants had a confirmed positive HIV PCR; 144 (54%) of these infants were female, 227 (85%) were born from high‐risk mothers and 18 (7%) were non‐Thai (Table 1). The median age of infants when blood was collected for the first‐positive PCR test declined from 75 days (interquartile range (IQR) 55 to 124) in the pre‐ACC period to 60 days (IQR 29 to 133) during the ACC (*P* < 0.001) (Table 1). Among the 267 infants diagnosed with HIV infection during the ACC, 60 (22%) were diagnosed with a birth specimen, 158 (59%) with the first PCR specimen collected after birth, 37 (14%) with the second PCR specimen and 12 (4%) with the third PCR specimen. One hundred and twenty‐six (29%) HIV‐positive infants were diagnosed before two months of age in the pre‐ACC period; this increased to 136 (51%) during ACC (Table 1).

Infants diagnosed during the ACC were more likely to initiate ART than infants diagnosed during the pre‐ACC (ACC 88%, pre‐ACC 56%; *P* < 0.001). A total of 81 (30%) infants diagnosed with HIV during the ACC started ART before two months of age, whereas 1 (<1%) infant diagnosed during the pre‐ACC period initiated ART before two months of age. Similar increases in ART uptake were seen at six months (ACC 184 (69%), pre‐ACC 52 (12%); *P* < 0.001), and one year (ACC 214 (80%), pre‐ACC 158 (37%); *P* < 0.001). Median age at ART initiation was 83 days (IQR: 48 to 160) in ACC compared to 282 days (IQR: 191 to 451) in the pre‐ACC period (*P* < 0.001). In ACC, 15 (6%) infants died and 11 (4%) were lost to follow‐up before ART initiation. The number of deaths before ART initiation was highest in the first year of ACC implementation (i.e. nine cases in 2015, four cases in 2016 and two cases in 2017). Median age at the time of HIV diagnosis among infants who died before ART initiation was 130 days (IQR 50 to 148) which was higher than the median age at the time of HIV diagnosis among all infants in ACC. Lost to follow‐up was highest in the Northern region, 7 (63%) infants including 3 (27%) non‐Thai citizens. Six (2%) infants were in follow‐up for ART initiation as of August 2018.

**Figure 4:**
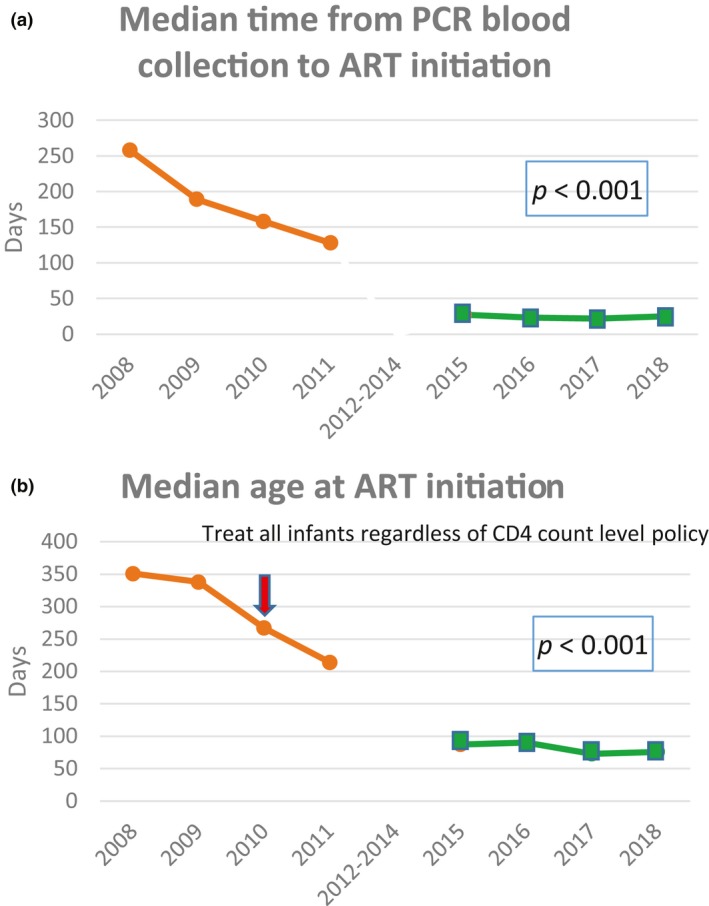
(a) Median time from diagnosed PCR blood collection to ART initiation. 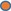 EID survey 2008 to 2011 (pre‐ACC): Diagnosed PCR positive is defined as the second‐positive PCR (confirmed) or the first PCR positive with no conflict result (probable). 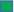 ACC 2015 to 2018: Diagnosed PCR positive is defined as first PCR positive (confirmed true positive with second PCR). (b) Median age at antiretroviral therapy (ART) initiation. 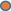 EID survey (pre‐ACC) 2008 to 2011. 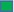 ACC 2015 to 2018.

Median time from blood collection to hospital notification of the positive PCR result in the ACC was 16 days (IQR: 11 to 22) (Table 2). Once the national laboratory CM was notified of the positive PCR result, the CM notified the responsible regional CM the same day or the next day. Median time from notification of the regional CM to date of ART initiation was six days (IQR: 1 to 16). Overall, the median time from blood collection to ART initiation declined from 168 days (IQR: 95 to 364) during the pre‐ACC period to 23 days (IQR: 15 to 34); *P* < 0.001 during ACC (Table 2, Figure 4a). The median age at ART initiation declined from 351 days in 2008 to 76 days in 2018 (Figure 4b) (*P* < 0.001).

**Table 2:** Time to ART initiation, HIV‐positive infants, Thailand, 2015 to 2018

Median time from birth to collection of first‐positive PCR	Median time from blood collection to hospital notification of positive PCR result	Median time from notification of +PCR result in routine system to notification of the central laboratory CM	Median time from notification of central laboratory CM of positive PCR to notification of regional CM	Median time from notification of regional CM to ART initiation
60 days (IQR 28 to 132)	16 days (IQR 11 to 22)	0 days (IQR 0 to 1)	0 days (IQR 0 to 1)	6 days (IQR 1 to 16)
Time from birth to ART initiation: 83 days (IQR 48 to 159)				
	Time from blood collection to ART initiation: 23 (IQR 15 to 34)			

ART, antiretroviral therapy; CM, case manager; IQR, Interquartile range; PCR, polymerase chain reaction.

## 4 Discussion

The Thailand MOPH in collaboration with U.S. CDC Thailand, the HIV‐NAT Research Center, universities and multiple hospitals in Thailand successfully organized and implemented an active case‐managed network (ACC). Since the network activities were launched in 2014, the coverage of EID and linkage of HIV‐positive infants to ART services has increased significantly. The time to identify HIV‐positive infants and initiate ART decreased and the number of new perinatal HIV infections declined 19. The ACC project provided healthcare providers who had little obstetric or paediatric experience with immediate access to national experts in EID, PMTCT and paediatric care. The median time from HIV diagnosis to ART initiation during ACC was six days. This reduction in the time to start ART is consistent with findings of a study in Eswatini using case management to improve linkage for HIV‐positive adults for early ART initiation 20 and supports WHO's recommendation for rapid ART initiation and a patient‐centred approach 21.

The successful implementation of the ACC network demonstrates that governments, research institutions, universities and healthcare institutions can collaborate, allocate resources and coordinate activities to achieve important public health and patient care goals such as increasing uptake of EID, using DBS for birth samples, and the early initiation of ART. The U.S. Department of Health and Human Services 22 and WHO 6 recommend that virological diagnostic testing at birth be considered for infants at high risk of perinatal HIV transmission. Implementing PCR at birth in Thailand is feasible because more than 98% of delivery in Thailand occur in health facilities 23. In addition, PCR at birth testing can be integrated with a routine neonatal screening programme in Thailand (i.e. congenital hypothyroidism and phenylketonuria) 24. Although the uptake of EID testing in the ACC model was high, preliminary data analysis of the national EID data 2018 found that about one‐fifth of HIV‐exposed infants who were tested received a PCR test at birth, but did not return for the second PCR (GAM report 2018, unpublished). This is consistent with a study in South Africa that reported implementing PCR at birth lowered the uptake of subsequent EID testing at six weeks of age 25. Details of Thailand's national EID programme, PCR at birth and outcomes of infants receiving birth testing will be reported in separate papers.

Although CMs worked diligently to follow‐up all HIV‐positive infants to initiate ART, a relatively high proportion of infants died (i.e. 5.6%) or were lost to follow‐up (i.e. 4.1%) before ART initiation, similar to findings in other countries 2, 26, 27, 28. We found that some of these HIV‐positive infants received late PCR testing. Some infants were lost to follow‐up before diagnosis and returned to care when they were sick. Exclusive replacement feeding with infant formula is recommended for all infants born to HIV‐positive mothers and the Thailand MOPH provides free infant formula to HIV‐exposed infants for 18 months 11, 13, nonetheless a study in Thailand found that 2.4% of HIV‐exposed infants were breastfed 29, 30. This is likely because mothers acquired HIV infection late antepartum or post‐partum and, the initial antenatal HIV testing result was negative, and these mothers did not receive PMTCT services 30 and were not aware of their HIV recent infection. Their babies did not receive PCR testing according to national recommendations and returned for care and received PCR testing late when they were sick. In addition, a report showed that triple‐neonatal prophylaxis regimen (6‐weeks of AZT/3TC/NVP) can delay the time of the first HIV PCR‐positive result 31. The majority of infant deaths occurred in the first year of ACC implementation. The number of deaths declined each year after ACC implementation. This may be because high‐risk infants who were infected in utero were diagnosed at birth with PCR testing and linked to HIV services including ART initiation early. Thailand's 2014 national policy for ART initiation transitioned from requiring two positive PCR results to one positive PCR. The median age of infant HIV diagnosis declined from 130 days during the pre‐ACC period to 60 days in infants diagnosed during the ACC programme. Non‐Thai infants, particularly those living in the Thai‐Myanmar border area in the Northern region of Thailand, were more likely to be lost to follow‐up compared to infants in other regions.

Data collected during the ACC project were used by WHO to validate the elimination of mother‐to‐child HIV transmission in Thailand in 2016 19, 23. The MOPH has integrated key components of the ACC programme into national guidelines and routine practice. Collection of a DBS at birth, particularly for HIV‐exposed infants with high risk for mother‐to‐child HIV transmission, was recommended in the national HIV treatment and care guidelines 2017 13, 14 and included in the national HIV benefits package in 2017. MOPH and U.S. CDC Thailand office conducted trainings in 2016 to 2017 to hand over the responsibilities of regional CMs to HIV coordinators of provincial hospital or provincial health offices in 77 provinces. Most ACC indicators are now routinely reported by healthcare providers in the National AIDS Program database.

This report has several limitations. The ACC network received reports of positive HIV PCR results from the national PCR laboratory and may not have captured infants diagnosed in private laboratories. We suspect that most if not all infants were diagnosed in the national laboratory 10. EID data included in the 2013 and 2014 Global AIDS Monitoring (GAM) reports 17, 32 were obtained from the National AIDS Program database that did not capture data on non‐Thai citizens whereas EID data in the 2015 to 2018 GAM reports 1 were from the National EID database which included reports from all Thais and non‐Thais living in Thailand. The number of HIV‐positive infants reported in this paper is drawn from programme PCR results and the denominator of MTCT rate is the number of infants who had PCR tests done. The actual MTCT rate in Thailand may be higher than the MTCT rate in this report because some HIV‐exposed infants may have died before diagnosis, may have been lost to follow‐up, and may have been infected post‐delivery by breast milk.

## 5 Conclusions

The ACC network has been successfully established in Thailand and integrated into routine medical services with strong collaboration from multiple partners. The initial results suggest the ACC network of laboratory notification using a social media platform among the ACC CMs and technical experts is promoting early ART initiation and shortening the time from blood collection to ART initiation. Yet, infant mortality and lost to follow‐up rates remain high. Additional work is needed to prevent HIV‐related infant mortality and to ensure follow‐up. This network may be adaptable to other settings where active case management can improve medical care and health outcomes.

## Competing interests

The authors declared no competing interests.

## Authors' contributions

RL participated in study design, project implementation, statistical analysis, interpretation of data and drafting and revision of the manuscript. PP, TP participated in study design, project implementation, data collection, interpretation of data and revision of the manuscript. PK, WP, TB, RH, AR, SB, TS, SO participated in study design, project implementation and data collection. MM participated in study design, statistical analysis, interpretation of data and revision of the manuscript. All authors reviewed and approved the final version of the manuscript.
